# Curvilinear pericallosal lipomas diagnosed incidentally during evaluation following trauma with corpus callosum abnormalities in two patients

**DOI:** 10.1259/bjrcr.20200081

**Published:** 2020-10-15

**Authors:** Mariam Kassimi, Hind Guerroum, Omar Amriss, Jihane Habi, Kaoutar Moutaouakil, Nabil Chikhaoui, Mohamed Mahi

**Affiliations:** 1 Department of Radiology, Faculty of Medicine, Mohammed VI University of Health Sciences/Cheikh Khalifa International University Hospital, Casablanca, Morocco; 2 20 Aout Radiology Department of the Ibn Rochd of Casablanca University, Casablanca, Morocco; 3 Department of Neurosurgery, Faculty of Medicine, Mohammed VI University of Health Sciences/Cheikh Khalifa International University Hospital, Casablanca, Morocco

## Abstract

P*ericallosal* lipomas are the most habitual location for an intracranial lipoma. They are fat-containing lesions arising from the interhemispheric fissure intimately related to the corpus callosum, which is often abnormal. They originate from aberrant differentiation of the persistent primitive meninx. Most Pericallosal lipomas are asymptomatic and come into clinical attention during neuroradiological investigations for other conditions. MRI is the modality of choice to characterize not only the extent of the lipoma but also the frequently associated agenesis/dysgenesis of the corpus callosum. Pericallosal lipomas can be divided into two groups: The Tubulonodular type and The curvilinear type. Curvilinear lipomas are less common than Tubulonodular. We report the clinical and radiological findings of curvilinear Pericallosal lipoma in two patients with corpus callosum abnormalities revealed incidentally during evaluation following trauma.

## Introduction

Pericallosal lipomas are rare, fat-containing asymptomatic lesions that are generally considered as congenital malformations. They generate approximately 0.1–0.5% of all intracranial Lesions. Two morphological types have been described: tubulonodular and curvilinear. The latter is mostly asymptomatic, sometimes presenting with headache, and has a low incidence of other concomitant anomalies.

In this study, we report clinical and radiological findings of two patients with curvilinear type Pericallosal lipoma associated corpus callosum abnormalities diagnosed incidentally during evaluation following trauma

## Case reports

### Case 1

**Figure 1.**
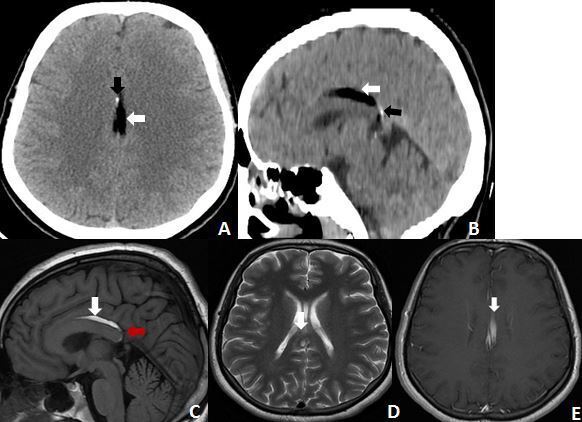
A, B: Brain CT axial and sagittal plans shows a homogeneous and well-circumscribed lesion in the interhemispheric fissure closely related to the corpus callosum with fat density (−80UH) (white arrow) and presence of calcification (black arrow). C, D: Pericallosal curvilinear lipoma. Sagittal T1W and axial T2W image demonstrate a linear hyperintense lesion (white arrow). Associated hypoplasia of the splenium of the corpus callosum is also seen(Red arrow). E: Axial T1W image after injection of gadolinium showing a linear hyperintense lesion in the interhemispheric fissure compatible with a curvilinear lipoma

A 32-year-old male was involved in a road traffic accident. He was brought to an emergency department, fully conscious and orientated but he was complaining headache. A brain CT was ordered that showed homogeneously and well-circumscribed lesion in the interhemispheric fissure intimately related to the corpus callosum with fat density (−80UH) and presence of a calcification consistent with pericallosal lipoma. The patient was discharged home with non-specific treatment. One month after, the patient consulted for persistent headaches. The neurological examination was unremarkable and the patient was subjected to MRI of the brain. It revealed a Pericallosal curvilinear lipoma shown as a linear hyperintense lesion surrounding the corpus callosum associated with hypoplasia of the splenium of the corpus callosum (Figure 1). It was decided to follow-up on the patient clinically.

### Case 2

**Figure 2.**
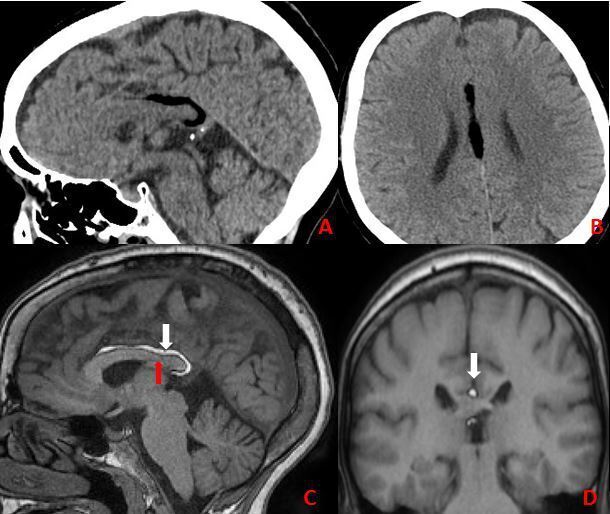
A, B: Brain CT sagittal and axial plans shows well-defined low-density in the pericallosal area and the interhemispheric fissure. C, D: Pericallosal curvilinear lipoma. Sagittal and coronal T1W MRI demonstrates a hyperintense lesion (white arrows) superior and posterior to the corpus callosum with mild dysgenesis of corpus callosum (red arrow).

A 68-year-old woman was referred to the neurosurgery department of our institution with a complaint of continuous headache due to a stairs fall taking place 2 months before. Her neurological examination was normal. Radiological evaluation included CT and MRI. CT scan of the brain was obtained that shows homogeneously hypodense midline lesion with a mean HU of −110 (fat). The MRI indicated for better study of the corpus callosum revealed a curvilinear-shaped area surrounding the body and the splenium of the corpus callosum, which appeared hyperintense in T1W images. Mild dysgenesis of the corpus callosum is also seen (Figure 2). This appearance was consistent with Lipoma of the Corpus Callosum. No surgical intervention was indicated.

## Discussion

Intracranial lipomas are rare congenital malformations, which presents 0.1–0,5% of all intracranial tumors.^1^

The most common location is the close vicinity of the corpus callosum (CC), hence the name pericallosal lipomas.^2^ Pericallosal lipomas arise from the interhemispheric fissure intimately related to the corpus callosum, which is often anomalous. The pathogenesis of a pericallosal lipoma is considered to be the consequence of abnormal resorption of the meninx primitive.^3^ When the meninx primitive remains longer, rather than being resorbed, it differentiates into lipomatous tissue.^4^ More than half of intracranial lipomas are associated with congenital malformations like agenesis or dysgenesis of the corpus callosum.^5^ Most pericallosal lipomas are asymptomatic and come into clinical attention during neuroradiological examinations for other conditions,^2^ which is the case in our patients. When symptomatic, however, the most habitual symptom is a headache.^6^ In some cases, pericallosal lipomas present with psychomotor retardation or epilepsy,^2^ due to associated anomalies of the corpus callosum.^6^ The imaging features of intracranial lipomas are well defined. On CT, they are observed as smooth-contoured, hypodense masses. CT densities range from − 40 to – 100^7^ ; In our two cases CT densities were measured at – 80 and − 90 HU. MRI is the most convenient modality for differential diagnosis (dermoid tumors mainly) and to reveal associated congenital malformations. The lipomas shape a homogenously hyperintense impression on T1W images. They do not enhance after i.v. gadolinium injection and they homogenously lose their intensity on fat suppression sequences.^8^ Pericallosal lipomas can be divided into two groups according to their shape: Curvilinear or Tubulonodular. It has been reported in the literature that curvilinear lipomas are less common than Tubulonodular.^3^ Tubulonodular is anteriorly located usually cylinder-shaped lipomas. These lipomas are frequently exceeding 2 cm in diameter and have a high incidence of corpus callosum dysgenesis, frontal lobe anomalies, and encephaloceles. Curvilinear lipomas are more extensive, posteriorly located generally present with a normal corpus callosum, and a low incidence of associated anomalies.^2,7,9^ In our two patients, we found corpus callosum abnormalities associated with curvilinear lipomas which is less frequent. We described hypoplasia of the splenium of the corpus callosum for the first case and mild dysgenesis in the body of the corpus callosum for the second one. However, this finding has been described in many recent studies (Yildiz and al in 24 patients) and (Yilmaz and al in 10 patients), who reports that the corpus callosum can be hypoplasic or mildly abnormal in curvilinear pericallosal lipomas. Truwit and Barkovich have shown in their series of 16 lipomas (including 11 Tubulonodular and five curvilinear lipomas) that the large anterior lipomas are more commonly associated with agenesis and the small posterior lesions with mild hypoplasia of the corpus callosum.^4^ This argument was supported by Tart Roger P^10^ and Quisling Ronald G who report that curvilinear lipomas are generally associated with a normal corpus callosum and have a low incidence of associated anomalies. When anomalies do appear, they tend to be less pronounced than with the Tubulonodular lipomas.^9^ As an explanation for this is that the curvilinear lipomas are developed after the closure of the anterior neuropore and thus cannot interfere with the development of this portion of the brain as do the earlier appearing Tubulonodular lipomas.^9^

Most pericallosal lipoma patients are asymptomatic. These lesions are generally discovered fortuitously and they grow very slowly. Therefore, Neurosurgical intervention is generally not required and it should be limited depending on the patient’s symptoms, surgical feasibility, and associated malformations. Imaging follow-up is not required too. Our two cases did not display surgical indications and they were observed in clinical follow-up.

## Conclusion

Pericallosal lipoma is of two types: Tubulonodular type and curvilinear type. Curvilinear lipomas are less common than Tubulonodular lipomas with a low incidence of associated anomalies of the corpus callosum. They are generally benign, self-limiting, or slow-growing and remain asymptomatic in the majority of the cases. The overall prognosis of pericallosal lipoma is good.

## Learning objectives

- Pericallosal lipoma is of two types: Tubulonodular type and curvilinear type.
- Curvilinear lipomas are less common than Tubulonodular lipomas with a low incidence of associated anomalies of the corpus callosum less pronounced.
- MRI is the most convenient modality for the diagnosis of pericallosal lipomas and the identification of associated congenital malformations.
